# Use of Grape Peels By-Product for Wheat Pasta Manufacturing

**DOI:** 10.3390/plants10050926

**Published:** 2021-05-06

**Authors:** Mădălina Iuga, Silvia Mironeasa

**Affiliations:** Faculty of Food Engineering, Ştefan cel Mare University of Suceava, 13 Universitatii Street, 720229 Suceava, Romania

**Keywords:** grape peels, pasta, wheat flour, fibers, antioxidants

## Abstract

Grape peels (GP) use in pasta formulation represents an economic and eco-friendly way to create value-added products with multiple nutritional benefits. This study aimed to evaluate the effect of the GP by-product on common wheat flour (*Triticum aestivum*), dough and pasta properties in order to achieve the optimal level that can be incorporated. Response surface methodology (RSM) was performed taking into account the influence of GP level on flour viscosity, dough cohesiveness and complex modulus, pasta color, fracturability, chewiness, cooking loss, total polyphenols, dietary fibers and resistant starch amounts. The result show that 4.62% GP can be added to wheat flour to obtain higher total polyphenols, resistant starch and dietary fiber contents with minimum negative effects on pasta quality. Flour viscosity, dough cohesiveness, complex modulus and pasta fracturability of the optimal sample were higher compared to the control, while chewiness was lower. Proteins’ secondary structures were influenced by GP addition, while starch was not affected. Smooth starch grains embedded in a compact protein structure containing GP fiber was observed. These results show that GP can be successfully incorporated in wheat pasta, offering nutritional benefits by their antioxidants and fiber contents, without many negative effects on the final product’s properties.

## 1. Introduction

Nowadays, consumer behavior is changing and functional foods with significant health benefits are gaining increasing attention. The food industry generates high amounts of by-products that may be considered an opportunity for a sustainable valorization in order to minimize the waste impact and to ensure environmental protection. Winery by-products’ conversion into value-added products is of high interest for producers, consumers and researchers. One method of valorization is the inclusion of byproducts in food products in order to increase their nutritional value. Pasta is consumed worldwide and can be considered a good matrix for bioactive compounds’ incorporation [1].

About 50% of the grape pomace is composed of grape peels (GP), depending on the grape variety and pedo-climatic conditions [2,3]. Some health problems such as cardiovascular diseases, stroke and some cancer types can be prevented by an adequate intake of fruits and vegetables due to their high amounts of bioactive compounds [4]. GP are a source of polyphenolic compounds and dietary fiber [5,6,7], components that can exert antioxidant an antimicrobial action [8,9]. The most important GP components with antioxidant characters are anthocyanins, hydroxycinnamic acids, catechins and flavonols, which can determine the inhibition of oxidative processes of low-density lipoproteins [10,11].

The potential applications of polyphenols and dietary fiber to preserve foods and prolong their shelf-life were demonstrated in some previous studies [12,13]. GP contain up to 60% (dm) dietary fiber, the insoluble fraction prevailing, followed by sugars, which can total up to 70%, depending on the vinery process applied [10,14]. Due to the essential role played by dietary fiber for human health, such as improvement of gastrointestinal activity, reducing glycemic responses and cholesterol levels in the blood [13], it is necessary to take alternative sources of dietary fiber to achieve the recommended consumption, which is about 25–30 g per day [1].

There are some studies revealing the possibility to create value-added products by incorporating grape by-products in bakery products or pasta [15,16,17,18]. Grape by-products can be used as semolina replacer in pasta with many positive effects on the technological properties, such as firmness and adhesiveness, but also on the physico-chemical and functional characteristics of the final product, such as increased total polyphenolics content and antioxidant activity, and lowering of the glycaemic index trough resistant starch content increase [1]. Simonato et al. [19] studied the effects of *Moringa oleifera leaf* powder on wheat fresh pasta and observed an increase of cooking loss and a reduced firmness along with nutritional value enhancement given by higher phenols and mineral contents. The addition of coconut by-products to wheat pasta led to lower firmness and color changes, while the fiber, protein and lipid contents increased [20]. Sobota et al. [21] underlined the possibility to increase durum wheat pasta fiber content by incorporating different vegetables powders (beet, carrot, kale), along with significant changes of color. According to the results presented by Xu et al. [22], the incorporation of apple pomace in noodles caused a cohesiveness and tensile strength reduction and a cooking loss increase, with the hardness and adhesiveness of the noodles not being changed, while gumminess, chewiness, and springiness recorded differences. Fortification of durum wheat pasta with onion skin by-products resulted in increased dietary fiber, ash, total phenolic compounds, flavonoids content and antioxidant activity, while cooking loss, water solubility index and redness were higher and the optimal cooking time lower [23]. Zarzycki et al. [24] showed that the addition of Moldavian dragonhead seeds residue in pasta resulted in higher nutritional value by increasing proteins, dietary fiber and mineral contents, without negative effects on the cooking and sensory characteristics of the pasta. Another study made by Simonato et al. [25] underlined the opportunity to increase total polyphenol contents and antioxidant capacity of pasta by supplementation with olive pomace. The authors found a decrease of rapidly digestible starch and an increase of slowly digestible starch, resistant starch, swelling index, water absorption, cooking loss and pasta firmness [25].

The addition of fiber-rich ingredients can have significant effects on dough rheological properties and on the final product texture, microstructure and color. The effects of grape by-products on the composite flour and final product properties are proportional to the addition level [3]. Mironeasa et al. [16] revealed some negative effects of GP on dough rheology caused by gluten dilution, which can be minimized by particle size reduction. Food texture, volume and color are strongly affected by high levels of grape by-products, Gaita et al. [17] suggesting that amounts up to 6% GP can be incorporated into pasta containing eggs without significant negative effects on the sensory acceptance. On the other hand, fortification of bakery and pasta products with grape by-products led to a nutritional value increase due to the intake of fiber and polyphenolics with antioxidant activity [4,5,15]. Grape peels’ phenolics, such as phenolic acids, tannins and flavonoids, could have reducing effects on starch digestibility due to their abilities to inhibit enzyme activity or by the formation of starch-polyphenol complexes with resistance to enzyme attacks [26]. Furthermore, polyphenols can slow down starch gelatinization via the interaction through hydrogen bonds with amylose molecules [27]. Saad et al. [28] found that wheat pasta dough rheological properties in terms of extensibility and water absorption increased, while elasticity decreased, when cucumber pomace was added. The mineral and polyphenols content of noodles were improved, while a reduction of protein and carbohydrate contents was observed [28]. 

In countries where durum wheat is not widely cultivated, common wheat usually represents the basic ingredient for pasta production. There are some studies revealing the possibility to use fiber and polyphenols-rich ingredients in pasta formulation, but to our knowledge, there are no studies revealing the effects of GP on common white wheat flour for pasta production. The approach of this study is complex, evidencing the technological, nutritional, molecular and structural changes of flour, dough and pasta. The knowledge of the interactions of grape peels with other biopolymers from wheat is very important for the development of novel pasta products. Thus, the aim of this study was to underline the impact of GP components on white wheat flour (WWF), dough and pasta properties, and to optimize the addition level in order to obtain the best product quality. Furthermore, a characterization of the optimum and control products was made.

## 2. Results

### 2.1. Diagnostic Checking of the Models

The ANOVA results for the model fitting presented in Table 1 show that all of the mathematic models chosen were significant and predicted accurately the responses, the *F*-value being significant (*p* < 0.01) in all cases and *R^2^* values being more than 0.76. 

Flour peak viscosity (η_max_), dough cohesiveness (Co) and boiled pasta chewiness (Ch) data were fitted to the quadratic model, which described 83%, 76% and 78% of the data variation, respectively. Dough complex modulus (G*), dry pasta chroma (C*), fracturability (F), cooking loss (CL) and polyphenolic content (TPC) results were fitted to the quartic model, with 91%, 91%, 98%, 97% and 96%, respectively, of the data variation being explained. The cubic model explained 98% of data variation for resistant starch content (RS), while the results for the dietary fiber content (TDF) were fitted to the fifth model, which explained 99% of the variation.

### 2.2. Effects of GP on Flour and Pasta Characteristics

A Supplementary Materials section containing the graphics for the variation of the responses with GP level is provided.

Peak viscosity increased with GP level increase (Figure S1), the biggest significant (*p* < 0.01) positive influence being obtained for the linear term Equation (1).

(1) $$\eta_{\max}\left(\mathrm{Pa}\cdot s\right)=0.42+0.14A^{**}-0.01A^{2}$$

where η_max_—peak viscosity, A—GP level, ** significant at *p* < 0.01.

An increase of G* as the GP level was higher was observed (Figure S2a), the linear and quadratic terms having significant influence (*p* < 0.05) on the response Equation (2).

(2) $$G^{*}\left(\mathrm{Pa}\right)=96083.29+23997.76A^{**}+38352.65A^{2}{}^{*}+808.26A^{3}-27020.94A^{4}$$

where G*—complex modulus, A—GP level, ** significant at *p* < 0.01, * significant at *p* < 0.05.

More cohesive dough was obtained as the GP addition level was higher (Figure S2b), the linear term having the greatest significant (*p* < 0.01) influence Equation (3). The negative effects of GP, which are a fiber-rich ingredient, were minimized because small particle size (<180 μm) was used.

(3) $$\mathrm{Co}=0.40+0.02A^{**}+0.01A^{2}$$

where Co—cohesiveness, A—GP level, ** significant at *p* < 0.01.

Pasta color was affected by GP incorporation, a significant (*p* < 0.01) decrease of C* being observed with the addition level increase (Figure S3a). The biggest negative influence was obtained for the linear term of the factor Equation (4).

(4) $$C^{*}=23.08-2.72A^{**}+1.42A^{2}-0.35A^{3}+0.03A^{4}$$

where C*—chroma, A—GP level, ** significant at *p* < 0.01.

Dry pasta fracturability expressed as the maximum force needed to break the sample can be an indicator of pasta resistance to transport and manipulation. The addition of GP caused an increase of F with the level increase (Figure S3b). The linear and cubic terms presented significant positive influence (*p* < 0.05), while the quadratic term had a negative effect on the response Equation (5).

(5) $$F\left(g\right)=3158.72+1837.63A^{**}-1052.11A^{2}{}^{**}+256.32A^{3}{}^{*}-19.84A^{4}{}^{*}$$

where F—fracturability, A—GP level, ** significant at *p* < 0.01, * significant at *p* < 0.05.

GP addition caused a CL rise with the level increase (Figure S4a), the linear, quadratic and quartic terms presenting significant (*p* < 0.05) influences based on Equation (6). An acceptable cooking loss value should be less than 12% [5].

(6) $$\mathrm{CL}\left(\%\right)=0.66+6.33A^{**}-3.02A^{2}{}^{*}+0.61A^{3}-0.04A^{4}{}^{*}$$

where CL—cooking loss, A—GP level, ** significant at *p* < 0.01, * significant at *p* < 0.05.

Pasta chewiness is expressed as the energy required to chop the sample until it is ready to swallow [29]. GP level increase caused a proportional decrease of pasta chewiness (Figure S4b), the highest influence being observed for the linear term Equation (7).

(7) $$\mathrm{Ch}=3696.18-287.20A^{**}+79.49A^{2}$$

where Ch—chewiness, A—GP level, ** significant at *p* < 0.01.

Resistant starch content was significantly influenced (*p* < 0.01) by the linear, quadratic and cubic terms in Equation (8). A rise in RS with GP addition level increase was observed (Figure S5a).

(8) $$\mathrm{RS}\left(\%\right)=4.49+0.34A^{**}-0.43A^{2}{}^{**}+0.50A^{3}{}^{**}$$

where RS—resistant starch content, A—GP level, ** significant at *p* < 0.01.

A significant positive influence (*p* < 0.01) was obtained for the linear term of GP level, while the quadratic and quartic terms exhibited a negative and significant (*p* < 0.05) effect on TPC response (Equation (9). TPC showed higher values with GP level increase (Figure S5b), as a result of polyphenols present in the added ingredient.

(9) $$\mathrm{TPC}\left(\%\right)=29.25+117.50A^{**}-55.362A^{2}{}^{*}+11.17A^{3}-0.77A^{4}{}^{*}$$

where TPC—total polyphenols content, A—GP level, ** significant at *p* < 0.01, * significant at *p* < 0.05.

GP are a rich source of soluble and insoluble dietary fibers, their incorporation in wheat pasta determining an increase of TDF with the level increase (Figure S5c). Significant positive influences (*p* < 0.01) were obtained for the linear, cubic and fifth terms, while the quadratic and quartic terms presented negative effects on the response (Equation (10)).

(10) $$\mathrm{TDF}\left(\%\right)=-4.58+9.17A^{**}-6.31A^{2}{}^{**}+2.01A^{3}{}^{**}-0.30A^{4}{}^{**}+0.02A^{5}{}^{**}$$

where TDF—total dietary fiber content, A—GP level, ** significant at *p* < 0.01.

### 2.3. Optimization of GP Level and Models Validation

To obtain the maximum nutritional benefits with minimum quality characteristics’ impairment, the optimization of GP level as a function of the considered responses showed that wheat flour can be supplemented with 4.62% GP (Table 2), with a desirability of 0.57. 

For the model’s validation, a pasta sample was made using the optimal level of GP that resulted after optimization. The responses were checked in triplicate and the experimental values were less than 5% different from the predicted ones (Table 2), except for chewiness, which was lower by 6.86% than the predicted value. Compared to the control, significantly (*p* < 0.01) higher G*, η_max_, dough Co, F, CL, RS, TPC and TDF contents were obtained, while C* and boiled pasta Ch were smaller (Table 2). Consequently, the nutritional and functional values of the optimized pasta were enhanced compared to the control and the quality parameters were kept. Even if higher CL was obtained (6.81%), the value was less than 12%, the limit recommended for acceptable pasta. The OGP sample presented a more cohesive, elastic and viscous dough, which was probably related to the higher resistance to break (F) of pasta, which was desirable. 

### 2.4. Determination of Control and Optimal Product Properties

#### 2.4.1. FTIR Analysis of Flours

**Figure 1:**
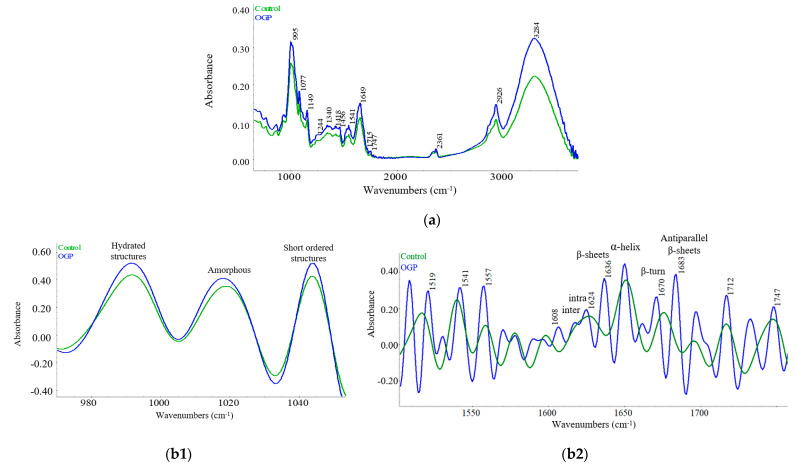
(**a**) Average spectra of optimal wheat-grape peels (OGP) and control samples in mid-infrared region; (**b1**) starch components’ deconvoluted spectra; (**b2**) protein and polyphenol components’ deconvoluted spectra.

FTIR analysis allowed the identification of changes in bonding and possible interactions between composite flour components, underlying the impact of GP addition to WWF. The representative spectra of OGP and control samples is presented in Figure 1 and shows the peaks of the functional groups and the vibration ways of the compounds. Several peaks were identified in the analyzed spectra (650–4000 cm^–1^) and were attributed to the molecular linkages of some chemical components such as starch, proteins and polyphenols.

The deconvoluted spectra in the range of 800–1300 cm^−1^ (Figure 1b1) showed the characteristics of starch grains. The amount of hydrated starch structures was identified at 995 cm^−1^, the amorphous starch at 1022 cm^−1^ and the short-ordered starch structures at 1047 cm^−1^ [30]. No significant changes (*p* > 0.05) were observed in starch structure between OGP and control samples (Table 3). The intermolecular associations were identified at 1613–1620 cm^−1^, the intramolecular associations at 1627–1635 cm^−1^, β-sheets structures at 1620–1644 cm^−1^, α-helix at 1650–1660 cm^−1^ and β-turn at 1660–1680 cm^−1^ [31,32]. The OGP sample presented higher inter- and intra-molecular associations compared to the control, which lacked absorbance for intramolecular associations. Significant lower (*p* < 0.01) α-helix conformations were identified for OGP compared to the control, while β-turn and antiparallel β-sheets structures were higher (Table 3). The presence of fibers could be observed from the peaks at 1149 and 1077 cm^−1^ [30], while the phenolic compounds could be identified at 1609–1608 and 1519–1516 cm^−1^ [33] (Figure 1b2) and 1747 cm^−1^.

#### 2.4.2. Pasta Chemical Properties

The chemical compositions of the OGP and control sample are presented in Table 3. 

GP addition to wheat flour caused a significant (*p* < 0.01) increase of the protein, lipid, ash and carbohydrate contents (Table 3) of pasta. The OGP sample presented higher radical scavenging activity (38.74%) compared to the control (20.15%). On the other hand, cooked pasta RDS significantly decreased (*p* < 0.01) when GP was added, while SDS increased compared to the control. 

#### 2.4.3. Microstructure Analysis

**Figure 2:**
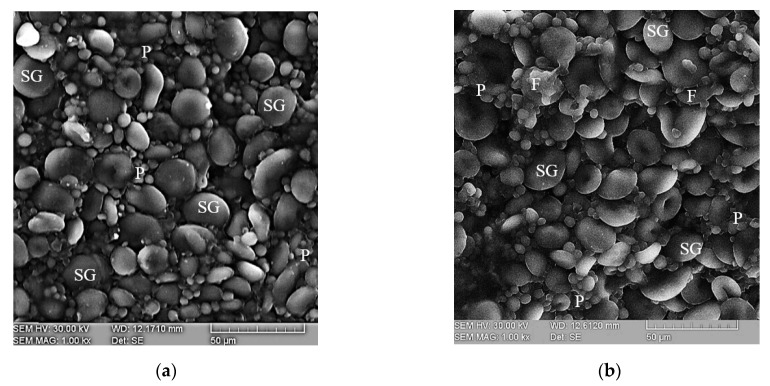
Dry pasta microstructure of (**a**) control and (**b**) optimal sample with grape peels (OGP): SG—starch grain; P—protein matrix; F—fiber.

Dry pasta microstructure analysis revealed a well-developed matrix comprised of a gluten network, which encompassed starch grains and fiber fractions (Figure 2). In both the control and OGP samples round and lenticular starch shapes with smooth surfaces were observed. The addition of GP resulted in a compact dough structure in which the proteins embedded the fine particles of fibers and starch grains. 

**Figure 3:**
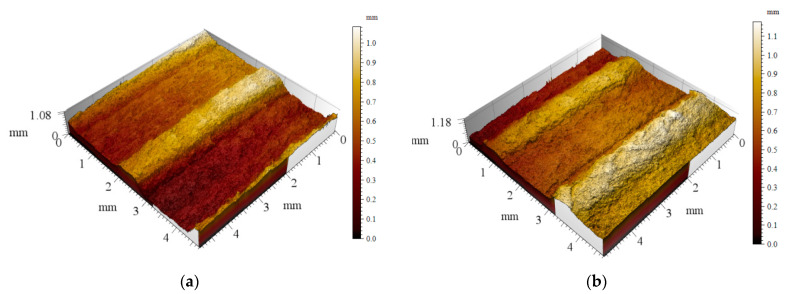
Three-dimensional dry pasta surface of (**a**) control and (**b**) optimal sample with grape peels (OGP).

Dough ingredients influenced the pasta extrusion process, which resulted in surface structure changes. The addition of GP caused a slight increase of pasta surface roughness, as can be seen in Figure 3. OGP pasta presented an uneven surface compared to the control. The alternation of high and low regions was given by the use of the *rigatoni* mold. 

## 3. Discussion

### 3.1. Effects of Grape Peels on Flour, Dough and Pasta Quality

GP caused an increase of flour slurry peak viscosity, a similar trend being reported by Mironeasa et al. [34] for wheat flour supplemented with GP and can be related to the affinity of GP for water, which may determine a viscosity increase. Probably, the pectin content of GP can be another factor responsible for the viscosity increase since it may assist starch swelling [35]. Masoodi et al. [36] also reported a reduction of wheat flour peak viscosity when more than 5% apple pomace was incorporated.

The dynamic complex modulus’ proportional increase with GP level could be explained by the strengthening effect of GP on the gluten network due to the presence of non-starch polysaccharides, tannins and polyphenols, which can form complexes with proteins [37]. Additionally, the oxidation of sulfhydryl groups to disulfides caused by the raised content of oxidizing compounds can possibly contribute to the complex modulus increase [3]. In this study, GP caused more cohesive dough. Mironeasa et al. [34] reported increased dough cohesiveness when small particle sizes of GP were used probably due to the ability of fine particles to better absorb water. GP fibers present many hydroxyl groups in their structure, which will determine higher interactions with water trough hydrogen bonds [38]. The sugars present in GP can also influence dough cohesiveness due to their water solubility [39]. Saad et al. [28] showed that pasta soft wheat dough supplemented with cucumber pomace rheological properties changed compared to the control, an increase of the extensibility and water absorption and a decrease of elasticity being observed.

Dry pasta chroma decreased as the amount of GP was higher. In the literature, Smith and Yu [40] reported higher b* (yellow nuance) and lower a* (red nuance) values of bread supplemented with grape pomace. High amounts of sugars found in GP can promote Maillard reactions and, along with the polyphenol’s presence, may determine C* decrease. Additionally, the natural pigments from GP are responsible for color intensity decrease [41]. Other studies reveled that the addition of by-products such as dragonhead seeds, coconut residue or *Moringa oleifera* L. leaf [19,20,24] produced significant changes in pasta color, depending on the pigments present in the ingredient added. The quality of the final product depends on the chemical composition of the ingredient added, soluble dietary fibers such as inulin and fructooligosaccharides playing an important role in pasta network strength [42]. Polyphenol interactions with proteins can determine stronger dough structure [18,43], leading to higher fracturability values. 

The higher cooking loss obtained in this study for pasta enriched with GP can be attributed to the polymer interactions in the gluten matrix and/or to the competition of proteins for water, leading to starch loss [44]. A similar trend of cooking loss was reported for fettuccini pasta supplemented with grape marc [5] and can be due to the dietary fiber content of the added ingredient. Xu et al. [22] reported also a proportional increase of pasta cooking loss with the addition level of apple pomace increase. Pasta texture can be a good predictor for consumer preferences. The results obtained showed a decreasing trend of pasta chewiness with GP level increase. The interference of GP components with starch, which led to a reduction of starch gelatinization during cooking, can be associated with lower chewiness [45]. The use of a small particle size (<180 μm) for GP flour can be an advantage for pasta quality by diminishing the negative impact on the texture. A study regarding the influence of apple pomace on wheat pasta showed that this fortification caused a decrease of pasta chewiness as the level was higher [22], similar trend being reported also by Chen et al. [33] for pasta enriched with more than 1% grape seeds.

The nutritional value of wheat pasta was increased by the incorporation of GP. The raised resistant starch content of pasta enriched with GP can be attributed to the interactions between polyphenols and starch [46,47] through non-covalent linkages formation. Furthermore, starch digestion diminution produced by the formation of linkages between polyphenols and starch can occur from the starch molecular structure modifications. A similar RS increase was reported by Simonato et al. [25] for pasta fortified with olive pomace. For instance, some studies revealed that these interactions can contribute to the ordered structure or development of starch crystallin areas, inducing RS formation [48,49]. On the other hand, the polyphenolics content of pasta increased with GP level increase. Gaita et al. [17] also reported higher polyphenolic contents for pasta supplemented with grape peels, the increase being directly proportional to the amount added. A study performed by Michalak-Majewska et al. [23] showed that the addition of onion skin powder caused an increase in total phenolic compounds, flavonoids content and antioxidant activity. The phenolic compounds present in grape by-products are highly accessible and available for metabolization in the human intestine [50]. In addition, the bioavailability polyphenols in pasta are directly related to the hydrogen, ionic, covalent, and hydrophobic interactions with proteins [3]. The addition of fiber-rich ingredients can be a useful technique to increase the nutritional value of the final product. Wheat pasta fiber content was enhanced by GP addition. Acun and Gül [51] also reported raised fiber content of cookies enhanced with seedless grape pomace. Similar to our study, Saad et al. [28] showed that the addition of cucumber pomace in soft wheat pasta induced the increase of fibers and polyphenol contents. Fibers are known to have some biological functions, such as activity against cancer, amelioration of gastrointestinal systems functioning, improvement of cardiovascular system activity, and decrease of cholesterol and glycaemia levels in blood [52]. Balli et al. [53] found that durum wheat tagliatelle with grape and olive pomace resulted in improved quality in terms of organoleptic and nutritional properties, with high levels of phenolic compounds and increased fibers content.

### 3.2. Control and Optimal Product Properties

In this study, the optimal amount of GP was found to be 4.62%; at this level acceptable technological characteristics and superior nutritional value compared to the control were obtained. The optimal sample with GP (OGP) and the control made of wheat flour were characterized.

FTIR spectra showed the interactions between components of the composite flours. The peak at 3284 cm^−1^ was attributed to the -OH stretching vibrations, while the peak at 2926 cm^−1^ was assigned to the C-H stretching and showed higher absorbances for OGP probably due to the phenolic compound’s presence [54]. The phenolic compounds found in GP have specific absorptions at 1712–1704 cm^−1^ corresponding to the carbonyl stretching and 1609–1608 and 1519–1516 cm^−1^ given by the stretching vibrations of C=C [55] (Figure 1b2). The galloyl group’s presence could possibly be observed at about 1747 cm^−1^ (Figure 1b2), which corresponds to the stretching vibrations of carbonyl groups (C=O) [54]. In the case of OGP, an increase of peak intensities at 1149 and 1077 cm^−1^ compared to the control could be due to the intake of small hemicellulose, cellulose and pectin [30] found in GP. Starch structure characterized by the vibrations given at 1022 cm^−1^ for amorphous and at 1047 cm^−1^ for short-ordered regions [30] was not significantly affected by the addition of GP. The absorption at 1700–1600 cm^−1^ was assigned to the Amide I fraction, which could offer information about the secondary structure of proteins [30]. GP addition in wheat flour caused inter- and intramolecular associations compared to the control, which lacked absorbance for these structures. Lower α-helix conformations were observed for OGP compared to the control, while β-turn and antiparallel β-sheet structures were present in higher proportion. These changes could be possibly related to the protein–polyphenols interactions in the dough matrix, a similar opinion being reported by Ertürk and Meral [56]. Sivam et al. [57] also reported lower α-helices and lower intermolecular associations when polyphenols were added to bread. Chen et al. [33] obtained a reduction of β-turn conformational composition of gluten proteins with grape seeds’ level increase, while β-sheet conformations increased. The α-helix secondary structures were dominant for both the OGP and control samples, similar results being obtained by Nawrocka et al. [58], who studied the influence of dietary fibers on gluten proteins. Dough rheological properties are directly influenced by protein secondary structure. Our results indicated a stronger and more cohesive dough structure of OGP, which can be related to the formation of protein–fiber complexes. The band at 1670 cm^−1^ is due to non-hydrogen linkages of carbonyl groups in the β-turn structures of proteins; when carbonyl groups are hydrogen bonded with other compounds, a decrease of their absorption band to smaller wavenumbers should be observed [58]. The results of the present study revealed that in the control sample the protein carbonyl groups were bonded trough non-hydrogen linkages, a fact evidenced by the presence of the 1670 cm^−1^ band, while in OGP the carbonyl groups would have formed hydrogen bonds with fibers or other GP components, a fact suggested by the shift to the left of the absorption band. This band shift could possibly be attributed also to the presence of polysaccharides such as pectin from GP [58]. On the other hand, the appearance of the absorption band at 1661 cm^−1^ in the OGP sample (Figure 1b2) could be related to the development of intramolecular and intermolecular hydrogen linkages between glutamine side chains and peptide groups, which was probably due to the influence of the GP fiber-rich ingredient [58]. The band at 1625 cm^−1^ observed for OGP can be associated with the hydrogen bonding of protein aggregates and/or of polypeptide chains complexed with phenolic compounds [59] from GP, as shown by their chemical composition.

GP raised the protein, lipid, ash and carbohydrate contents of pasta due to their intake of nutrients. Compared to our study, Saad et al. [28] showed that cucumber pomace addition in soft wheat noodles increased the mineral and polyphenols content, but decreased protein and carbohydrates. The addition of GP caused higher radical scavenging activity compared to the control, in agreement with the polyphenols content, which presented a raised value for pasta samples with GP. Gaita et al. [17] also reported higher antioxidant capacity of pasta enriched with grape peels. Cooked pasta RDS significantly decreased (*p* < 0.01) when GP were added, while SDS increased compared to the control. Similar trends of RDS and SDS were reported by Simonato et al. [25] when wheat pasta was fortified with olive pomace, which may be due to the starch content reduction caused by the addition of the fiber-rich ingredient. These results could be related to the starch–polyphenol interactions that may occur during pasta making. It has been demonstrated that polyphenols can reduce starch digestion rates due to their interactions trough hydrophobic forces with amylose and the linear fraction of amylopectin and/or to the inhibition effects on enzymes [49]. On the other hand, fibers from GP could compete with starch granule for water, reducing starch gelatinization, thereby causing starch digestibility limitation [25].

GP incorporation resulted in a compact dough structure in which the proteins embedded the fine particles of fibers and starch grains. Similar results were presented by Tolve et al. [1] for durum wheat pasta with grape pomace. The denser gluten network of pasta with GP is probably due to the intake of protein, cellulose and polysaccharides, which can act as fillers in the gluten matrix, while the polyphenols present in GP may interact with gluten proteins to support the formation of a gluten matrix, similar observations being made by Chen et al. [33] for pasta enriched with 1% grape seeds. Huang et al. [60] also reported a dense structure of wheat noodles formed of starch granules embedded in a developed fiber matrix. Pasta surface roughness was higher when GP was added. This increase could be possibly due to the difference in the water absorption capacity of the composite flour compared to the control, a difference caused by the presence of fibers from GP [34]. According to the study of Chen et al. [33], the incorporation of more than 3% grape seeds in wheat noodles induced the appearance of jagged edges and uneven mesh structure of pasta caused by the non-gluten components. 

## 4. Materials and Methods

### 4.1. Materials

The research was conducted on 650 white wheat (*Triticum aestivum*) flour (WWF) type from the 2019 harvest, provided by Dizing S.R.L. (Brusturi, Neamt, Romania). Wheat flour chemical components reported to dry matter were: lipids content of 1.11 ± 0.03%, protein content of 14.41 ± 0.21%, carbohydrates 81.26 ± 0.16%, ash content of 0.60 ± 0.05%, moisture of 14.01 ± 0.16%, total polyphenols content of 105.59 ± 4.42 μg GAE/g. The falling number was 404 ± 1.73 s, wet gluten content was 29.75 ± 0.15%, dry gluten was 10.04 ± 0.13%, gluten deformation index was 6.17 ± 0.29 mm and the water absorption capacity was 59.54 ± 0.31%. Grape pomace from the Fetească Regală variety was provided by Iași Research and Development Center for Viticulture and Vinification and was dried in a convection oven at 50 °C for 18 h. Grape peels were manually separated, ground and sieved to obtain the particle size of <180 μm. Grape peels contained 2.50 ± 0.19% lipids, 9.85 ± 0.05% proteins, 25.25 ± 0.05% fibers, 55.73 ± 0.20% carbohydrates, 4.47 ± 0.03% ash, 8.00 ± 0.08% moisture and 1448.69 ± 15.39 μg GAE/g polyphenols, 0.40 ± 0.01% pectin reported to dry matter (dm) and a water absorption capacity of 271.10 ± 4.34%. Wheat flour was sieved before mixing in order to achieve a particle size of <300 μm. Composite flours were mixed for 15 min in a Yucebas Y21 machine (Izmir, Turkey). 

### 4.2. Pasta Processing

Pasta dough was mixed in a Kitchen Aid mixer (Whirlpool Corporation, Benton Harbor, MI, USA) by adding the amount of water calculated in order to obtain 40% moisture. Pasta modeling was performed after 15 min of the dough resting at room temperature, by using a rigatoni mold of the Kitchen Aid machine. Pasta drying was performed in a convection oven, according to the method described by Bergman et al. (1994) as follows: 30 min drying in open air at room temperature, followed by a first step of drying for 60 min at 40 °C, a second for 120 min at 80 °C and a third for 120 min at 40 °C. 

### 4.3. Evaluation of GP Effects on WWF and Pasta Quality

#### 4.3.1. Flour Pasting Properties

Flour pasting behavior in terms of peak viscosity η_max_ (Pa·s) was simulated on a dynamic rheometer Thermo-HAAKE, MARS 40 (Karlsruhe, Germany) with a Peltier temperature controller, by using a cup-cylinder geometry. The method described by Ahmed et al. [61] was adapted as follows: the slurry was kept at 50 °C for 60 s, followed by a heating at 95 °C for 222 s, keeping at 95 °C for 210 s, cooling at 50 °C for 228 s and keeping at 50 °C for 120 s. 

#### 4.3.2. Fundamental Rheological Behavior

Laminated dough samples were rested for 30 min for internal strain removal and tested for linear viscoelastic region (LVR) by using a Thermo-HAAKE, MARS 40 (Karlsruhe, Germany). A frequency sweep test was performed for the determination of the complex modulus G* (Pa). For this purpose, the sample was placed between the parallel plates at a 3 mm gap and a vaseline layer was applied on the exposed edges for moisture loss prevention. The frequency was varied from 0.1 to 20 Hz, at a strain of 15 Pa that was in the LVR.

#### 4.3.3. Dough Texture

For dough cohesiveness (Co) determination, a texture analysis was performed by using a Perten TVT-6700 texturometer (Perten Instruments, Hägersten, Sweden). A double compression was applied to dough balls of 50 g weight, at 50% height, a speed of 5.0 mm/s and a trigger force of 20 g [62].

#### 4.3.4. Dry Pasta Color

Pasta chroma C* Equation (11) of the CIE Lab system was determined by reflectance by using a Konica Minolta CR-400 (Tokyo, Japonia) colorimeter.

(11) $$C^{*}=\sqrt{a^{*2}+b^{*2}}$$

where C*—chroma, a*—red or green nuance, b*—yellow or blue nuance.

#### 4.3.5. Pasta Fracturability

Pasta fracturability as the maximum force F (g) required to break a dry pasta piece was determined with a Perten TVT-6700 texturometer (Perten Instruments, Sweden). An aluminum break rig set adjusted to 13 mm width was used, the test speed being set to 3 mm/s and the trigger force to 50 g [62].

#### 4.3.6. Total Polyphenolics Content 

The extracts were prepared according to the method described by Melilli et al. [63]. An amount of 2 g of grinded uncooked dry pasta was extracted with 20 mL of methanol 80% (*v/v*) in a sonication bath at 37 °C and 45 Hz for 40 min, and then the mix was filtered. Total polyphenols content (TPC) (μg GAE/g dm) of dry pasta was evaluated by using the Folin–Ciocalteu method [64]. The extract was diluted in a ratio of 1:4 with distillated water and mixed with Folin–Ciocalteu reagent (1N) and sodium carbonate 20% (*v/v*) and left to rest in darkness for 40 min. The absorbance was read at 725 nm on an UV–VIS–NIR Shimadzu 3600 (Tokyo, Japan) spectrophotometer. TPC was calculated from a calibration curve (*R*^2^ = 0.99) made with gallic acid (GAE).

#### 4.3.7. Total Dietary Fiber Content

For dietary fiber content TDF (% dm) estimation, a FOSS 6500 NIR (FOSS NIRSystems, Silver Springs, FL, USA) infrared spectrometer was used. Uncooked dry pasta samples were ground before the analysis and the spectra were collected at room temperature. For calibration, off the shelf INGOT commercial calibrations (AUNIR, Towcester, UK) were used. Standard materials provided by AUNIR were used for bias corrections [65]. 

#### 4.3.8. Pasta Cooking Behavior

The loss of solids CL (%) during pasta cooking was determined gravimetrically by evaporation of the water that resulted after boiling 10 g of pasta in 200 mL of water for the optimum cooking time previously established [66]. 

#### 4.3.9. Boiled Pasta Texture

Pasta chewiness Ch (J) was determined by double cycle compression on one piece of pasta by using a Perten TVT-6700 device (Perten Instruments, Sweden) equipped with a 35 mm cylinder probe, at 50% of the sample height, a test speed of 5.0 mm/s and a trigger force of 20 g [62]. 

#### 4.3.10. Rapid Digestible Starch (RDS), Slowly Digestible Starch (SDS) and Resistant Starch (RS) Contents

The international AOAC 2017.16 method was used for RDS, SDS and RS determination from boiled pasta, by using Megazyme kit. After 20 min (for RDS), 120 min (for SDS) or 240 min (for RS) of sample digestion with α-amylase and amyloglucosidase the reaction was stopped and the mix was digested again with amyloglucosidase. The resulting glucose was determined by using GOPOD reagent and reading of the absorbance at 510 nm. The results were reported as percent to dry matter. 

### 4.4. Optimization of Grape Peels Level and Models Validation

GP level optimization was performed by using the trial version of Design Expert software (Stat-Ease, Inc., Minneapolis, USA). For this purpose, the multiple response optimization and the desirability function were used and the goals were selected as follows: G*, Co, C*, F, TPC, TDF and RS were maximized, CL and Ch were minimized, and η_max_* was kept in range. The experimental design matrix containing mean values of three replications of the responses is presented in Table 4.

For model validation, pasta was made using the optimal level of GP obtained and the response values were checked. The real values of the optimum sample characteristics were compared to the control made of untreated wheat flour. For the evaluation of differences among the predicted and the experimental results of the optimal solution, and among the optimal and control sample, *t* tests for two samples (*p* < 0.05) were performed.

### 4.5. Determination of Control and Optimal Product Properties

#### 4.5.1. Chemical Composition and Antioxidant Activity

The chemical composition was determined according to the Romanian and International standard methods and the results are reported to dry matter: moisture (SR EN ISO 712/2010), ash (SR ISO 2171/2002), protein (SR EN ISO 20483/2007) and lipids (SR 91/2007). TDF was determined by NIR (as described in Section 4.3.6) and the carbohydrates were calculated by difference. 

The radical scavenging activity (%) was evaluated by using 2, 2-di (4-tert-octylphenyl)-1-picrylhydrazyl (DPPH). The extract prepared as described in Section 4.3.5 (0.5 mL) was diluted with methanol 80% (0.5 mL) and mixed with 5 mL of DPPH. After resting 30 min in the darkness the absorbance was read at 517 nm on an UV–VIS–NIR Shimadzu 3600 (Tokyo, Japan) spectrophotometer [67]. 

#### 4.5.2. ATR-FT-IR Analysis of Flour

FT-IR spectra of the flours were collected in triplicate in the range of 650 to 4000 cm^−1^ by using a Thermo Scientific Nicolet iS20 (Waltham, MA, USA) spectrometer, at a resolution of 8 cm^−1^ by 64 scans. The fractions of amide I (1835–1585 cm^−1^), polyphenols (1516–1747 cm^−1^) and starch (800–1300 cm^−1^) were identified, the data being processed with OMNIC software. The starch, polyphenols and protein structures were assessed according to previous studies [31,68,69,70]. Fourier deconvolution was applied in order to characterize starch and protein structures. 

#### 4.5.3. Microstructure

Electronic scanning microscopy was employed for flour microstructure evaluation with a VEGA II LSH scanning electronic microscope (Tescan, Brno, Czech Republic). The acceleration tension was 30 kV and the magnification 1000×, the samples being fixed with adhesive carbon bands.

Pasta surface structure analysis was performed on a Mahr CWM100 microscope (Gottingen, Germany). The data collected were processed with Mountain Map trial version software (Digital Surf, Besançon, France).

### 4.6. Statistical Analysis

All of the analyses in the present study were performed in triplicate. XLSTAT for Excel 2021 version (Addinsoft, New York, USA) was used for statistical analysis of the data. In order to evaluate the significant differences (*p* < 0.05) among samples the *t*-test was used.

For the assessment of GP addition effects on WWF and pasta quality, mathematical modeling of the results was carried out by using the trial version of Design Expert software (Stat-Ease, Inc., Minneapolis, USA), by applying response surface methodology (RSM). The effect of GP level factor on the responses (η_max_, G*, Co, C*, Ch, F, CL, TPC, TDF and RS) was observed by using a D-optimal design with one factor varied at six levels (1, 2, 3, 4, 5, 6%) and three replications. The model’s fitting was evaluated through a sequential Fisher test, coefficients of determination (*R*^2^) and adjusted coefficient of determination (*Adj.-R*^2^), at a 95% confidence level. The most suitable model was selected according to the highest *Adj.-R*^2^ value.

## 5. Conclusions

The addition of grape peels increased the amounts of nutrients in wheat pasta, especially fibers, resistant starch and polyphenols, which are compounds with many health benefits. The optimal quantity of grape peels that can be added to wheat flour was found to be 4.62%. Dough cohesiveness, dry pasta fracturability and cooked pasta chewiness were enhanced compared to the control, while the cooking losses were within acceptable limits (<12%). Starch digestibility of pasta was positively influenced by grape peels, higher resistant starch, slowly digestible starch and lower rapid digestible starch being obtained. Interactions between wheat flour and grape peels were shown trough FT-IR analysis, significant changes of protein structures being observed. A compact microstructure of pasta was noticed at 4.62% addition of small particle sizes (<180 μm). Thus, this study can be helpful for both processors and consumers, underlying the opportunity to enhance the nutritional value of wheat pasta by incorporating grape peels, with benefits for human health and environmental waste management. This research was performed on a laboratory scale, and thus more investigations at the industrial level are needed. 

## Figures and Tables

**Table 1:** ANOVA results for the fitted models for different characteristics of flour, dough and pasta.

Response	Model	*F*-Value	*p*-Value	*R* ^2^	*Adj.-R* ^2^
η_max_ (Pa·s)	quadratic	36.23	<0.01	0.83	0.81
G* (Pa)	quartic	31.27	<0.01	0.91	0.88
Co	quadratic	24.14	<0.01	0.76	0.73
C*	quartic	32.72	<0.01	0.91	0.88
F (g)	quartic	179.30	<0.01	0.98	0.98
Ch (J)	quadratic	26.38	<0.01	0.78	0.75
CL (%)	quartic	91.58	<0.01	0.97	0.96
RS (%)	cubic	181.00	<0.01	0.98	0.97
TPC (μg GAE/g)	quartic	85.84	<0.01	0.96	0.95
TDF (%)	fifth	1503.06	<0.01	0.99	0.99

η_max_—peak viscosity, G*—complex modulus, Co—cohesiveness, C*—chroma, F—fracturability, Ch—chewiness, CL—cooking loss, RS—resistant starch, TPC—total polyphenols content, TDF—total dietary fiber.

**Table 2:** Confirmation of the optimized parameters and control sample characteristics.

Parameter	OGP			Control
	Predicted Value	Experimental Value	Relative Deviation * (%)	
A-GP (%)	4.62 ± 0.00	4.62 ± 0.00	-	-
η_max_ (Pa·s)	0.83 ± 0.06 ^x^	0.85 ± 0.06 ^xa^	2.35	0.42 ± 0.02 ^b^
G* (Pa)	113,595.55 ± 6604.45 ^x^	113,733.33 ± 950.44 ^xa^	0.12	51,170.00 ± 1822.44 ^b^
Co	0.41 ± 0.01 ^x^	0.41 ± 0.01 ^xa^	0.00	0.36 ± 0.01 ^b^
C*	19.07 ± 0.40 ^x^	19.26 ± 0.06 ^ya^	0.99	21.89 ± 0.06 ^b^
F (g)	5432.15 ± 106.15 ^x^	5659.67 ± 159.22 ^xa^	4.02	4207.33 ± 123.18 ^b^
Ch (J)	3583.12 ± 116.08 ^x^	3353.11 ± 162.77 ^xa^	−6.86	4910.27 ± 72.29 ^b^
CL (%)	6.81 ± 0.25 ^x^	7.03 ± 0.24 ^xa^	3.13	5.53 ± 0.19 ^b^
RS (%)	4.60 ± 0.10 ^x^	4.79 ± 0.01 ^ya^	3.97	2.58 ± 0.10 ^b^
TPC (μg GAE/g)	141.48 ± 4.21 ^x^	144.99 ± 2.78 ^xa^	2.42	106.75 ± 4.18 ^b^
TDF (%)	1.43 ± 0.03 ^x^	1.38 ± 0.03 ^xa^	−3.62	0.02 ± 0.00 ^b^

OGP—optimal formulation of wheat flour with grape peels, A—GP (grape peels) level, η_max_—peak viscosity, G*—complex modulus, Co—cohesiveness, C*—chroma, F—fracturability, Ch—chewiness, CL—cooking loss, RS—resistant starch, TPC—total polyphenols content, TDF—total dietary fiber, means in the same row followed by different letters (x–y for differences among predicted and observed values, a–b for differences between OGP and control) are significantly different (*p* < 0.05), * relative deviation = [(experimental value − predicted value)/experimental value] × 100.

**Table 3:** Optimal and control product properties.

Parameter	OGP	Control
Intermolecular associations (%)	1.71 ± 0.09 ^a^	0.00 ± 0.00 ^b^
Intramolecular associations (%)	4.75 ± 0.05 ^a^	0.00 ± 0.00 ^b^
β-sheets (%)	13.17 ± 0.44 ^a^	13.23 ± 0.58 ^a^
α-helix (%)	17.25 ± 0.71 ^a^	26.67 ± 1.89 ^b^
β-turn (%)	13.22 ± 0.39 ^a^	2.23 ± 0.59 ^b^
Antiparallel β-sheets (%)	19.74 ± 0.93 ^a^	5.44 ± 0.85 ^b^
Hydrated crystallin starch structure (%)	32.16 ± 0.37 ^a^	34.66 ± 1.90 ^a^
Short-ordered crystallin starch structure (%)	27.26 ± 0.20 ^a^	27.25 ± 0.20 ^a^
Amorphous starch structure (%)	34.26 ± 0.13 ^a^	34.00 ± 0.31 ^a^
Protein content (% dm)	14.29 ± 0.10 ^a^	13.93 ± 0.09 ^b^
Lipid content (% dm)	0.21 ± 0.02 ^a^	0.13 ± 0.02 ^b^
Ash (% dm)	0.80 ± 0.08 ^a^	0.63 ± 0.01 ^a^
Carbohydrates (% dm)	83.07 ± 0.22 ^a^	85.28 ± 0.11 ^b^
Radical scavenging activity (%)	38.74 ± 1.14 ^a^	20.15 ± 0.26 ^b^
RDS (% dm)	54.38 ± 0.24 ^a^	69.78 ± 0.69 ^b^
SDS (% dm)	19.61 ± 0.95 ^a^	17.35 ± 0.20 ^b^

OGP—optimal formulation of wheat flour with grape peels, RDS—rapid digestible starch, SDS—slowly digestible starch, dm—dry matter, a–b means in the same row followed by different letters are significantly different (*p* < 0.05).

**Table 4:** The effects of GP on the responses used in the experimental design.

GP (%)	η_max_ (Pa·s)	G* (Pa)	Co (adim.)	C* (adim.)	F (g)	CL (%)	Ch (J)	RS (% dm)	TPC (μg GAE/g dm)	TDF (% dm)
1.00	0.56 ± 0.03	82,936.67 ± 4914.90	0.39 ± 0.01	21.46 ± 0.63	4181.00 ± 103.00	4.55 ± 0.30	4034.97 ± 9.10	3.20 ± 0.08	102.00 ± 7.11	0.02 ± 0.01
2.00	0.62 ± 0.05	90,176.67 ± 7961.22	0.39 ± 0.01	20.94 ± 0.47	4357.00 ± 199.02	5.43 ± 0.18	3975.14 ± 168.70	4.10 ± 0.05	118.72 ± 2.76	0.35 ± 0.05
3.00	0.74 ± 0.12	96,045.00 ± 1395.00	0.40 ± 0.00	20.58 ± 0.49	4519.00 ± 139.54	5.72 ± 0.17	3700.57 ± 140.00	4.31 ± 0.09	124.91 ± 3.36	0.60 ± 0.00
4.00	0.81 ± 0.04	99,103.33 ± 7081.74	0.41 ± 0.01	19.65 ± 0.26	4998.33 ± 15.50	6.09 ± 0.31	3621.45 ± 100.87	4.57 ± 0.01	129.12 ± 2.00	1.05 ± 0.05
5.00	0.83 ± 0.16	122,600.00 ± 6080.60	0.42 ± 0.01	18.78 ± 0.23	5685.67 ± 54.78	7.28 ± 0.36	3589.50 ± 119.78	4.67 ± 0.04	149.27 ± 2.02	1.30 ± 0.00
6.00	0.87 ± 0.07	131,893.33 ± 8144.26	0.43 ± 0.01	18.54 ± 0.25	5961.00 ± 17.00	7.99 ± 0.14	3478.03 ± 102.51	4.88 ± 0.16	157.02 ± 4.38	1.50 ± 0.00

GP—grape peels level, η_max_—peak viscosity, G*—complex modulus, Co—cohesiveness, C*—Chroma, F—fracturability, Ch—chewiness, CL—cooking loss, RS—resistant starch, TPC—total polyphenols content, TDF—total dietary fiber.

## Data Availability

Not applicable.

## Supplementary Materials

The following are available online at https://www.mdpi.com/article/10.3390/plants10050926/s1, Figure S1: Effect of GP level on peak viscosity (ηmax); Figure S2: Effect of GP level on dough: a. complex modulus (G*), b. cohesiveness (Co); Figure S3: Effect of GP level on dry pasta: a. chroma (C*), b. fracturability (F); Figure S4: Effect of GP level on pasta: a. cooking loss (CL), b. chewiness (Ch); Figure S5: Effect of GP level on pasta: a. resistant starch content (RS), b. total polyphenols content (TPC), c. total dietary fiber (TDF).
