# Supplementary material for: Use of Grape Peels By-Product for Wheat Pasta Manufacturing
Source: Plants (Basel). 2021 May 6;10(5):926. doi: 10.3390/plants10050926 (PMC8148588; doi:10.3390/plants10050926)
Supplement: Supplementary file 1 [file plants-10-00926-s001.zip › plants-1191333-supplementary.pdf]

SUPPLEMENTARY MATERIAL

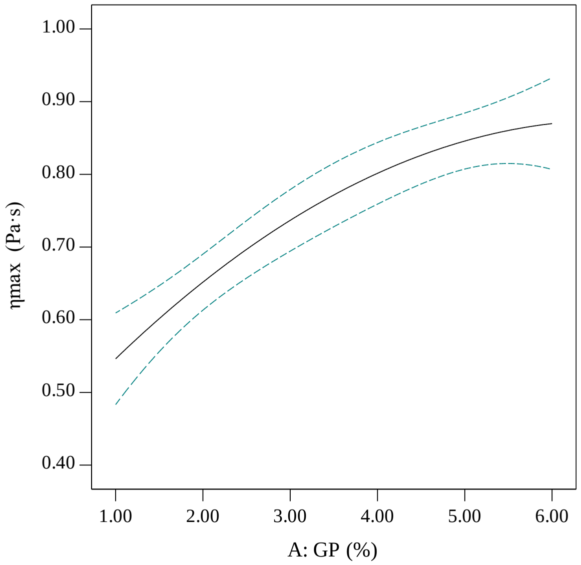

Figure S1. Effect of GP level on peak viscosity (η_{max})

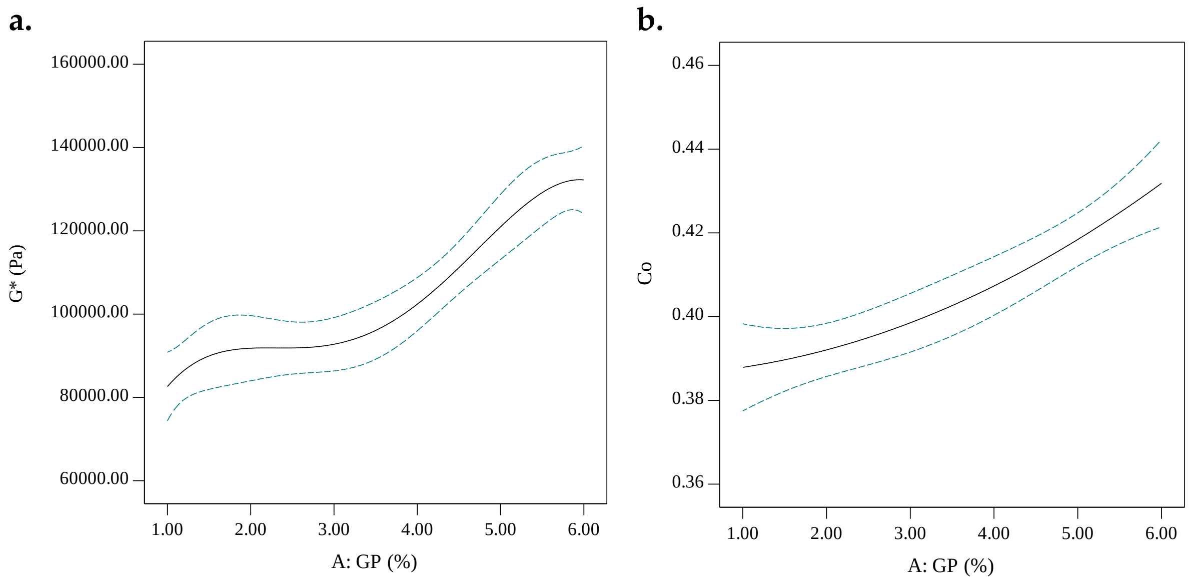

Figure S2. Effect of GP level on dough: a. complex modulus (G*), b. cohesiveness (Co)

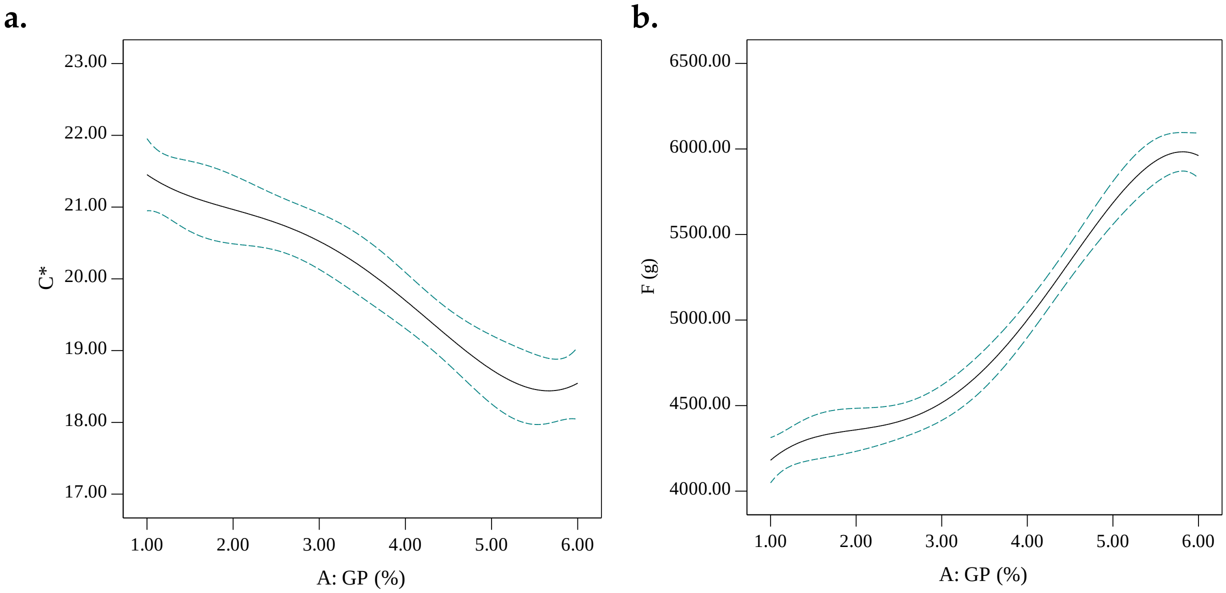

Figure S3. Effect of GP level on dry pasta: a. Chroma (C*), b. fracturability (F)

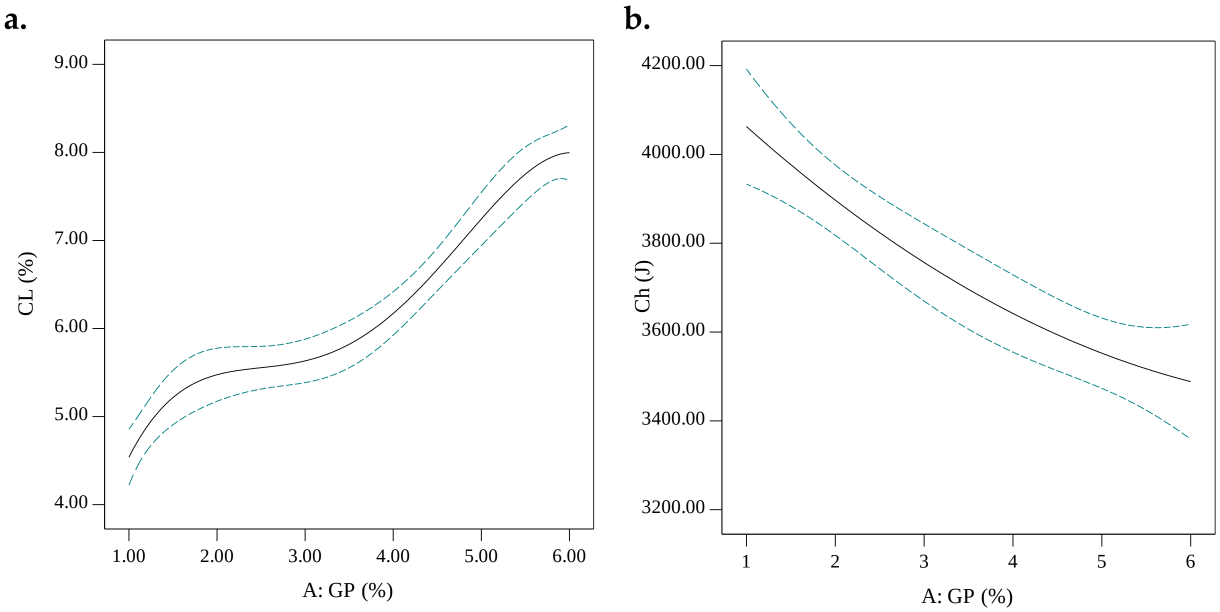

Figure S4. Effect of GP level on pasta: a. cooking loss (CL), b. chewiness (Ch)

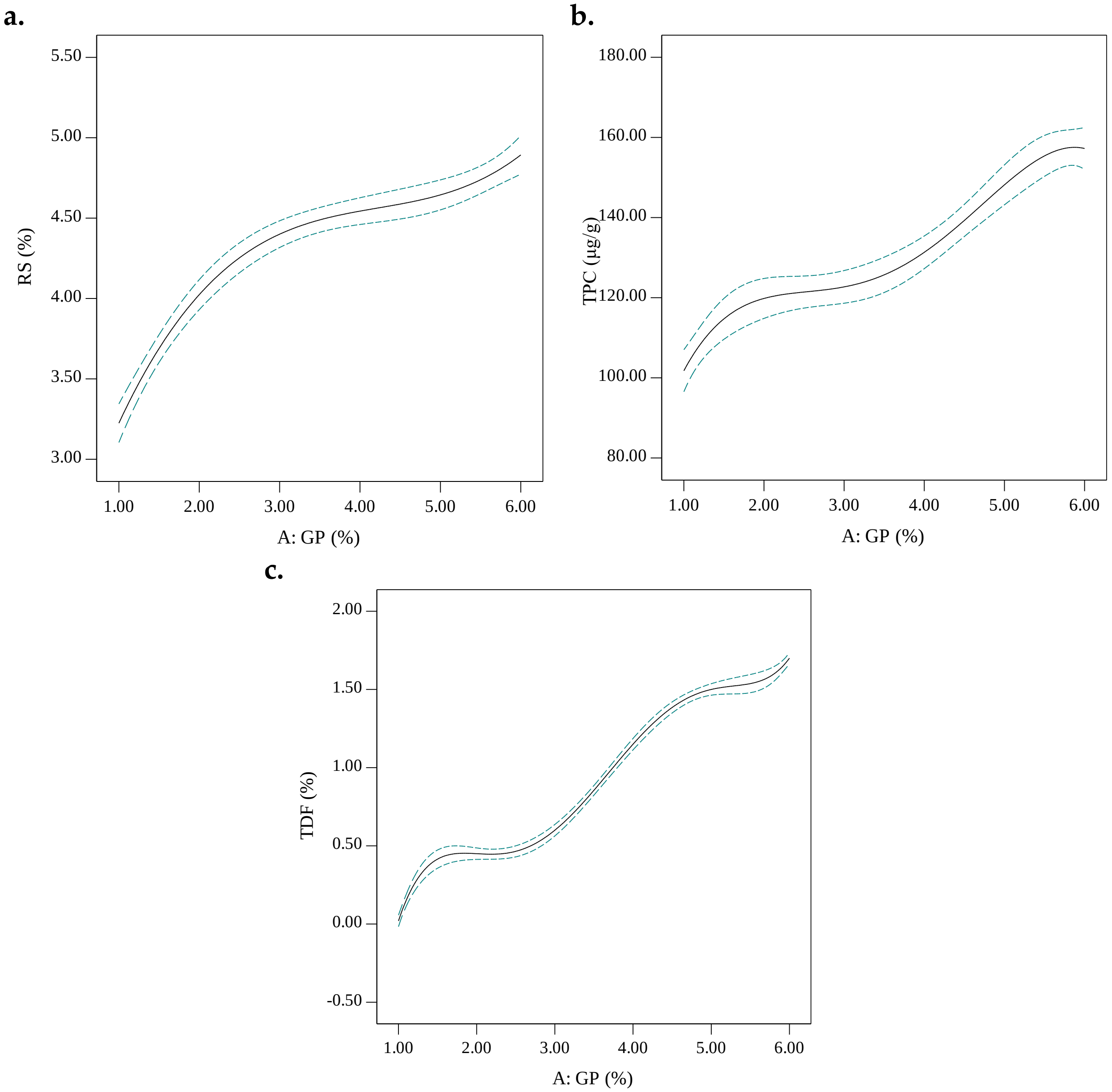

Figure S5. Effect of GP level on pasta: a. resistant starch content (RS), b. total polyphenols content (TPC), c. total dietary fiber (TDF)
